# AA147 ameliorates post-cardiac arrest cerebral ischemia/reperfusion injury through the co-regulation of the ATF6 and Nrf2 signaling pathways

**DOI:** 10.3389/fphar.2022.1028002

**Published:** 2022-11-23

**Authors:** Zhu Yuan, Liping Lu, Yingtao Lian, Yuanrui Zhao, Tingting Tang, Song Xu, Zhun Yao, Zhui Yu

**Affiliations:** ^1^ Department of Critical Care Medicine, Renmin Hospital of Wuhan University, Wuhan, China; ^2^ Central Laboratory, Renmin Hospital of Wuhan University, Wuhan, China; ^3^ Department of Anesthesiology, Renmin Hospital of Wuhan University, Wuhan, China

**Keywords:** cardiac arrest, AA147, neuroprotection, ischemia/reperfusion injury, endoplasmic reticulum stress, oxidative stress, ATF6 (activating transcription factor 6), Nrf2 (nuclear factor E2-related factor 2)

## Abstract

Ischemia/reperfusion caused by cardiac arrest (CA) disturbs endoplasmic reticulum (ER) homeostasis and redox balance in neurons. AA147, originally developed as a pharmacologic activator of the activating transcription factor 6 (ATF6), can protect multiple tissues from ischemia/reperfusion injury (IRI) by decreasing reactive oxygen species (ROS) and restoring ER function. However, it is unclear whether pharmacologic treatment of AA147 could ameliorate post-CA cerebral IRI and whether it is associated with proteostasis regulation and anti-oxidative stress mechanism. In the present study, mice were subjected to 9 min-CA surgery followed by cardiopulmonary resuscitation (CPR). AA147 or vehicle was administered 1 day before the operation and 15 min after the return of spontaneous circulation. We found that AA147 restored neurological function and reduced dead neurons in mice suffering from CA. Moreover, AA147 inhibited CA/CPR-caused neuronal apoptosis and ER stress, indicated by reduced TUNEL-positive neurons, surged expression of Bcl-2/Bax, and down expression of cleaved caspase-3, caspase-12, C/EBP homologous protein (CHOP). The expression of ATF6 and its regulated gene glucose-regulated protein 78 (GRP78) increased significantly after the administration of AA147, suggesting the activation of the ATF6 pathway. In addition, AA147 also alleviated the upsurge of the ROS generation and MDA levels as well as increased SOD activity, accompanied by enhancement of the nuclear factor E2-related factor 2 (Nrf2) and its modulated heme-oxygenase-1 (HO-1) expressions. Cotreatment of AA147 with inhibitors of the ATF6 or Nrf2 significantly suppressed AA147-dependent reductions in ROS scavenging and neuronal death after CA/CPR. The results suggested that AA147 could confer neuroprotection against post-CA cerebral IRI through inhibition of oxidative stress along with ER stress-associated apoptosis, which is attributed to the coregulation of both ATF6 and Nrf2 signaling pathways activity. Our findings support the potential for AA147 as a therapeutic approach to improve post-CA brain injury.

## Introduction

Cardiac arrest (CA) is correlated with high mortality and morbidity globally (Gowens et al., 2022), usually accompanied by severe neurological disability even after successful resuscitation (Tsao et al., 2022). Due to the slightest ischemia tolerance, the brain is the most susceptible to ischemia/reperfusion injury (IRI) among organs in CA (Geocadin et al., 2019). The primary neurological damage is triggered by ischemia during CA. In neuronal cells, ischemia-caused deficiency of oxygen and glucose delivery leads to depletion of adenosine triphosphate (ATP), which results in cellular excitotoxicity and disrupts calcium homeostasis. Secondary damage occurs during cardiopulmonary resuscitation (CPR) and sustains after resuscitation. Energy failure and calcium overload cause mitochondrial dysfunction and reactive oxygen species (ROS) overproduction, followed by disruption of endoplasmic reticulum (ER) proteostasis and neuroinflammation (Sekhon et al., 2017; Perkins et al., 2021; Sandroni et al., 2021). The double whammy drives massive neurons to death, leaving poor neurological outcomes or even death to patients. However, currently, there is a lack of effective drugs for post-CA brain injury.

It has been generally acknowledged that ER stress is an essential step in the onset and progression of IRI in various organs resulting from CA, especially cerebral IRI (Li and Yang, 2021). Protein homeostasis or proteostasis is modulated predominantly by the ER in the cell, as nearly a third of proteins are synthesized and folded in the ER (Wang and Kaufman, 2016). Insufficient ATP production, calcium overload, and ROS excess caused by ischemia/reperfusion lead to a significant increase of unfolded and misfolded proteins within the ER of the neuronal cell. And then, the unfolded protein response (UPR) is triggered to restore the proteostasis of the ER (Gulyaeva, 2015; Han et al., 2021; Li and Yang, 2021). The UPR consists of three branches, activating transcription factor 6 (ATF6), inositol-requiring enzyme 1 (IRE1), and protein kinase RNA-like ER kinase (PERK). When activated, they can reduce new protein synthesis and entry into the ER while enhancing ER’s ability to fold and degrade unfolded proteins, thereby preserving ER proteostasis and promoting cell survival (Hetz et al., 2020). Furthermore, ATF6 has recently been reported to be capable of regulating antioxidant genes and diminishing ROS generation, thus shielding cardiomyocytes from IRI (Jin et al., 2017). However, whether ATF6 could exert a similar effect on neurons and the relevant mechanisms remains to be elucidated.

Specific activation of the UPR branches with pharmacologic tools appears promising for attenuating IRI. AA147 is a novel small molecular compound identified by the high-throughput screen, selectively activating the ATF6 pathway (Plate et al., 2016). A recent study has shown that AA147 mitigated cardiac IRI caused by acute myocardial infarction, reducing oxidative stress and improving proteostasis in an ATF6-dependent manner (Blackwood et al., 2019). However, a subsequent study suggested that AA147 protects neuronal cells against glutamine-induced oxidative stress injury primarily by activating the nuclear factor E2-related factor 2 (Nrf2)-mediated signaling pathway, with activation of ATF6 contributing modestly (Rosarda et al., 2021). Nrf2 acts as a critical transcription regulator to modulate multiple downstream antioxidant enzymes and counteract oxidative stress caused by ischemia/reperfusion (Yamamoto et al., 2018; Shen et al., 2019). It is unclear whether pharmacologic treatment of AA147 could strengthen the antioxidant system by upregulating the Nrf2 signaling pathway to alleviate post-CA/CPR cerebral IRI.

In the present study, we administered AA147 in mice subjected to CA/CPR and then evaluated the neurological injury and examined the change in related signaling pathways. Here, we show that AA147 protects against CA/CPR-induced cerebral IRI by activating the ATF6 arm of UPR and Nrf2 signaling pathways, attenuating oxidative stress and ER stress-related apoptosis in neurons. The results demonstrated that AA147 is a potential compound to ameliorate post-CA brain injury through the coordinated regulation of the ATF6 and Nrf2 signaling pathways.

## Materials and methods

### Animals

Adult male C57BL/6 mice (8–10 weeks old, 25 ± 3 g) were obtained from China Three Gorges University and housed in the Animal Experiment Center of Renmin Hospital of Wuhan University. All experimental procedures were performed in adherence to the Guide for the Care and Use of Laboratory Animals (NIH, United States) and approved by the Institutional Animal Care and Use Committee of Renmin Hospital of Wuhan University (No. WDRM 20171204).

### Drug administrations and experimental design

**FIGURE 1:**
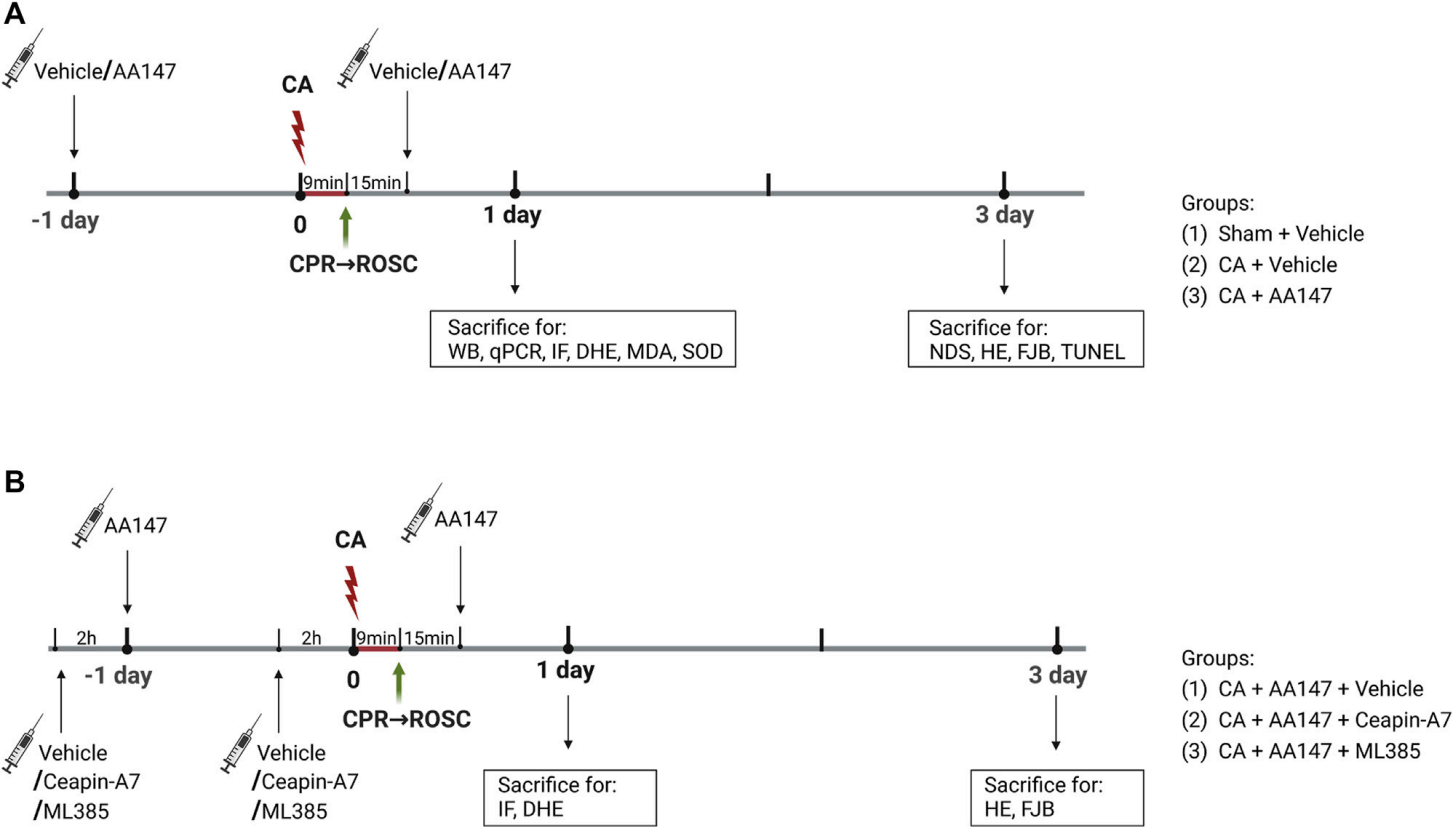
Experimental design and timeline. **(A)** Mice were subjected to a 9-min cardiac arrest surgery followed by cardiopulmonary resuscitation (CA/CPR) or a sham operation. Vehicle or AA147 (ATF6 activator; 2 mg/kg) was administered intraperitoneally 1 day before surgery and intravenously 15 min after the return of spontaneous circulation (ROSC) once again. The impact of AA147 on CA/CPR-induced oxidative stress and ER stress-related apoptosis as well as activation of the ATF6 and Nrf2 signaling pathways was detected at 1 day after CA/CPR. The effect of AA147 on neuroprotection after CA/CPR was evaluated at 3 days after CA/CPR. Groups: (1) Sham + Vehicle; (2) CA/CPR + Vehicle; (3) CA/CPR + AA147. **(B)** Ceapin-A7 (ATF6 inhibitor; 10 mg/kg) or ML385 (Nrf2 inhibitor; 30 mg/kg) was administered intraperitoneally 2 h before administration of AA147. The role of AA147-induced activation of the ATF6 or Nrf2 in the neuroprotective effect of AA147 against post-CA/CPR cerebral ischemia/reperfusion injury was assessed at respective timepoint. Groups: (1) CA/CPR + AA147 + Vehicle; (2) CA/CPR + AA147 + Ceapin-A7; (3) CA/CPR + AA147 + ML385.

In the first stage, mice were randomly divided into three groups: 1) sham + vehicle; 2) CA/CPR + vehicle; 3) CA/CPR + AA147. Compound AA147 (ATF6 activator; 2 mg/kg; MCE, cat. No. HY-124293) was administered intraperitoneally 1 day before surgery and intravenously 15 min after the return of spontaneous circulation (ROSC) once again. The effect of AA147 on ER stress and oxidative stress as well as activation of the ATF6 and Nrf2 signaling pathways, were detected at 1 day after CA/CPR. Neurological deficit scores, behavioral tests, and histopathological staining were used to evaluate the neuroprotective effect of AA147 treatment at 3 days after CA/CPR. In the second stage, to explore the underlying mechanism of AA147-dependent neuroprotection against IRI, Ceapin-A7 (ATF6 inhibitor; 10 mg/kg; MCE, cat. No. HY-108434) or ML385 (Nrf2 inhibitor; 30 mg/kg; MCE, cat. No. HY-100523) was administered intraperitoneally 2 h before the administration of AA147. Mice were randomly assigned into the following three groups: 1) CA/CPR + AA147 + vehicle; 2) CA/CPR + AA147 + Ceapin-A7; 3) CA/CPR + AA147 + ML385. Brain tissues were collected to detect the activation of specific signaling pathways and ROS activity levels at 1 day after CA/CPR. Neuronal death in the hippocampus of mice was assessed at 3 days after CA/CPR. The schematic diagram of the experimental design is illustrated in Figure 1. The overall assignment of mice for specific experiments is shown in Supplementary Table S1.

### Cardiac arrest and cardiopulmonary resuscitation animal model

CA/CPR operation was performed as previously described (Liu et al., 2018). Briefly, tracheal intubation was given after anesthesia induction (4%–5% isoflurane), and anesthesia was maintained in mice by mechanical ventilation with 1.0%–1.5% isoflurane. The electrocardiogram (ECG) was monitored throughout the procedure. A rectal temperature probe, a heat lamp, and a heating pad were used to maintain the mice’s body temperature at 37.0°C ± 0.2°C until ROSC. The right jugular vein was performed catheter insertion for the delivery of drugs. Heparin (50 μl, 5 U) was given after the right jugular vein was cannulated, and then 300 μl blood was drawn. CA was induced by infusing 30 μl of 0.5 M potassium chloride (KCL), and immediately the ventilation ceased. The cessation of spontaneous breath and ECG confirmed CA. At 3 min after CA, the blood was reinfused slowly. Resuscitation was initiated at 9 min following CA onset by resuming mechanical ventilation with 100% O_2_ and infusing a dose of epinephrine (100 μl, 32 μg/ml) with continuous pumping at 20 μl/min till 150 μl, as well as chest compression. ROSC was characterized by sustained ECG sinus rhythms. Resuscitation was stopped if ROSC was not attained in 4 min, and the mouse was removed from the experiment (Supplementary Table S1). When mice regained adequate spontaneous breath, they were disconnected from the ventilator and transferred into a thermal incubator (32.0°C) to recover for 2 h before returning to their home cages.

### Neurologic scores

Neurologic dysfunction was evaluated by a 9-point scoring system (Shen et al., 2018). Mice were scored based on their performance on the vertical screen, horizontal bar, and rope (0–3 points for each test), and then the overall neurologic score was obtained. 9 points represent normal, whereas 0 points indicate severe impairments.

### Open field test

Mice were gently put inside the open field box to move unrestrictedly for 10 min with an overhead camera videotaping them. The ANY-Maze software was used to record and analyze the indicators, including action time and travel distance.

### Y-maze test

Spatial recognition memory was accessed by the Y-maze test. The Y-maze apparatus was split into three arms converging into a central area: the A, B, and C arm. In the first stage, mice were given 10 min to explore the A and B arms while the C arm was clogged. During the second stage, all three arms were available for mice to explore for 15 min. The two stages were conducted at a one-hour interval. The ANY-Maze system was utilized to record and analyze the results.

### Hematoxylin-eosin staining

Neuronal aberrant morphology was assessed using Hematoxylin-eosin (HE) staining. Mice were anesthetized and transcardially perfused with cold PBS, followed by 4% paraformaldehyde. After being fixed in 4% paraformaldehyde overnight at 4°C, the brains were dehydrated, embedded in paraffin, and sliced into 3 μm coronal slices. The sections were deparaffinized and blocked, stained with hematoxylin and eosin, and then visualized by Olympus microscopy.

### Terminal deoxynucleotidyl transferase dUTP nick end labeling assay

Apoptosis-positive neurons were determined by the Terminal deoxynucleotidyl transferase dUTP nick end labeling (TUNEL) assay. We used an *In Situ* Cell Death Detection Kit (Roche, Switzerland) to perform the assay in compliance with the manufacturer’s instructions. Paraffin-embedded sections of mouse brain tissue prepared previously were deparaffinized, followed by exposure to proteinase K for rupture of the cell membrane, and then incubated with TUNEL mixture for 1 h at 37°C. After DAPI had been applied to label the nucleus, we used anti-fluorescence quenching sealing tablets to seal the slides. Finally, slices were viewed under the Olympus microscope, with images captured. Apoptotic cells of the hippocampus were calculated in each slide.

### Fluoro-Jade B staining

Fluoro-Jade B (FJB) staining was used to investigate the degeneration/death of neurons. The brain sections prepared previously were incubated in 0.06% potassium permanganate for 10 min, followed by 20 min in 0.004% Fluoro-Jade B solution (Millipore, Merck, Germany) at 37°C. After washing and drying, the slides were mounted with coverslips and viewed under the Olympus microscope. The number of FJB-positive cells in the hippocampal CA1 area was manually counted.

### Immunofluorescent staining

The coronal brain sections were deparaffinized and boiled with citrate buffer for 10 min for antigen retrieval. Then the sections were permeated with 0.5% Triton X-100 (prepared with PBS) for 20 min at room temperature. After being blocked in blocking buffer for 1 h at 37°C, the slices were treated with the primary antibodies overnight at 4°C. Then the sections were incubated with secondary antibodies conjugated to Alexa Fluor 488 or Alexa Fluor 594 for 1 h at 37°C. Fluorescence staining was observed under the Olympus microscope and analyzed by ImageJ software. The primary antibodies were listed as follows: ATF6α (1:200, Santa Cruz, cat. sc-166659); Nrf2 (1:200, Abcam, cat. ab62352).

### Detection of reactive oxygen species generation

The ROS generation was measured by Dihydroethidium (DHE) staining. The brains of previously harvested mice were frozen in liquid nitrogen immediately before embedding in OCT and slicing up to 4–5 μm fresh frozen coronal sections. After being incubated with DHE staining solution (1:500; Sigma, cat. D7008) for 30 min at 37°C away from light, the sections were imaged by the Olympus microscope. Then the fluorescence intensity was analyzed by ImageJ software.

### Malondialdehyde assay and superoxide dismutase assay

Malondialdehyde (MDA) assay was performed to measure lipid peroxidation. The level of superoxide dismutase (SOD) activity indirectly reflects the ability of cells to remove ROS. The hippocampal tissues were isolated from the brain, homogenized with lysis buffer, and centrifuged to get the supernatant. The MDA and SOD activity levels were detected using commercially available MDA assay kits (Jiancheng Biotech, Nanjing, China) and SOD assay kits (Jiancheng Biotech, Nanjing, China) according to the manufacturer’s protocols.

### Reverse transcription and real-time PCR

Total RNA of the hippocampal tissues was extracted using TRIzol reagent (Accurate Biology, Hunan, China) with the purity and concentration of RNA tested by micro-spectrophotometer (NanoDrop, Thermo Fisher, United States). Then RNA (1 μg each sample) was reverse transcribed into cDNA using Evo M-MLV RT Kit with gDNA Clean (Accurate Biology, Hunan, China). On a LightCycler 480 real-time PCR equipment, quantitative real-time PCR was conducted to evaluate relative mRNA levels using the SYBR® Green Premix Pro Taq HS qPCR Kit. The resulting Ct values were normalized to GAPDH, and the relative gene expression was determined using the 2 ΔΔCt method. Results were denoted as fold change relative to the control group. Primers used for the experiment are listed as follows: GAPDH: forward (5′-GTC​TCC​TCT​GAC​TTC​AAC​AGC​G-3′) and reverse (5′-ACC​ACC​CTG​TTG​CTG​TAG​CCA​A-3′); ATF6: forward (5′-GCG​GAT​GAT​AAA​GAA​CCG​AGA​G-3′) and reverse (5′-ACA​GAC​AGC​TCT​TCG​CTT​TG-3′); GRP78: forward (5′-CGT​ATG​TGG​CCT​TCA​CTC​CT-3′) reverse (5′-TTT​CTT​CTG​GGG​CAA​ATG​TC-3′); Nrf2: forward (5′-TCT​TGG​AGT​AAG​TCG​AGA​AGT​GT-3′) and reverse (5′-GTT​GAA​ACT​GAG​CGA​AAA​AGG​C-3′); HO-1:forward (5′-GAT​AGA​GCG​CAA​CAA​GCA​GAA-3′) and reverse (5′-CAG​TGA​GGC​CCA​TAC​CAG​AAG-3′).

### Western blot

The hippocampus tissues were resuspended in a lysis buffer containing 1% protein inhibitor and then homogenized by an ultrasonic cell processor. The lysates were boiled at 95°C for 5 min and centrifuged at 14,000 rpm for 10 min. Then we mixed the supernatants with isopycnic 2x loading buffer, followed by boiling and centrifugation. The total proteins were loaded on the SDS-PAGE, separated by electrophoresis, and then transferred to a 0.22 μm PVDF membrane. After blocking in 5% skim milk for 1 h at room temperature, the membranes were incubated with the primary antibody overnight at 4°C. After incubating with fluorescent second antibodies for 1 h at room temperature, the membranes were scanned by Odyssey CLx near-infrared laser imaging system. The resulting bands were analyzed using ImageJ software. The primary antibodies we used were as follows: ATF6α (1:500, Santa Cruz, cat. sc-166659); GRP78 (1:500, CST, cat. ^#^3177); caspase-12 (1:500, CST, cat. ^#^35965); CHOP (1:500, CST, cat. ^#^2895); Bcl-2 (1:500, CST, cat. ^#^3498); Bax (1:500, CST, cat. ^#^2772); caspase-3 (1:500, CST, cat. ^#^14220); cleaved-caspase-3 (1:500, Abcam, cat. ab32042); Nrf2(1:500, Abcam, cat. ab62352); Keap1 (1:500, Santa Cruz, cat. sc-365626); HO-1 (1:500, Abcam, cat. ab.68477); catalase (1:500, Abmart, cat. T56783S).

### Statistical analysis

Values obtained from the three or more independent experiments were represented as mean ± SD of individual data points and statistically analyzed using Graphpad Prism 9.0 Software. We used one-way analysis of variance (ANOVA) followed by Tukey’s multiple comparisons to analyze the significance of different groups. A *p* value <0.05 were considered statistically significant.

## Results

### AA147 attenuated cerebral IRI induced by CA/CPR

**FIGURE 2:**
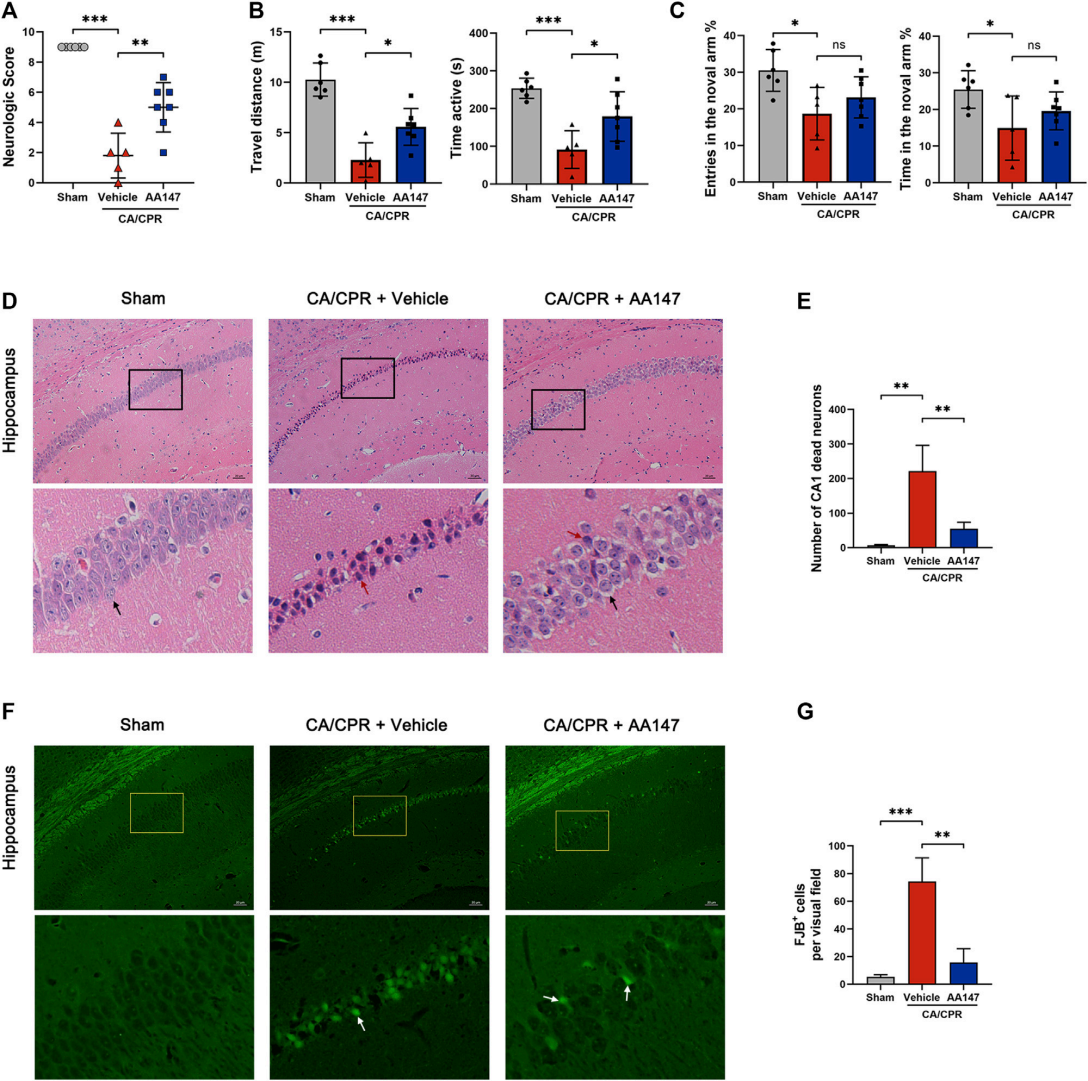
AA147 improved neurological outcomes and decreased neuronal death post-cardiac arrest and cardiopulmonary resuscitation (CA/CPR). Mice were subjected to sham or CA/CPR surgery, with vehicle or AA147 administered 1 day before the surgery and 15 min after the return of spontaneous circulation (ROSC). The effect of AA147 on improving neurological function and reducing neuronal death was evaluated at 3 day after CA/CPR. AA147-treated mice scored significantly higher in the neurologic scoring system: the 9-point scoring system (vertical screen, horizontal bar, and rope) **(A)**, and had longer travel distance and active time in the open field test **(B)**. However, there was no significant difference in the improvement of cognitive function between group CA + Vehicle and CA + AA147, presented in Y-maze. **(C)** Data are presented as mean ± SD (*n* = 5–7). **(D)** Representative HE staining images of the hippocampal cornu ammonis 1 (CA1) region and **(E)** counts of dead CA1 neurons. Black arrows indicate the normal pyramidal cells with intact morphology, while red arrows show the dead neurons with shrunken profiles and pyknotic and deeply-stained nuclei. Scare bar = 20 μm. Data are displayed as mean ± SD (n = 3). **(F)** Representative images of FJB staining and **(G)** the number of FJB^+^ neurons in the hippocampal CA1 region of each group. White arrows show the FJB^+^ neurons stained with green fluorescence, indicating neuronal death. Scare bar = 20 μm. Data are displayed as mean ± SD (*n* = 3). *p < 0.05. **p < 0.01. ***p < 0.001.

To investigate whether AA147 could exert a protective effect on the murine brain after CA/CPR, we used the 9-point scoring system, the open field, and the Y-maze test to evaluate the neurological outcomes at 3 days after CA/CPR. Compared with the sham group, mice experiencing CA/CPR performed dreadfully in scoring, while treatment with AA147 before and during CA/CPR improved cerebral functional recovery (Figure 2A). Moreover, the mice treated with AA147 exhibited better performance in the open field, as presented in Figure 2B. However, the tendency of the Y-maze test did not reach statistical significance (Figure 2C). To further assess the cerebral histopathological injury, we performed HE staining and FJB staining at 3 days after CA/CPR. Figures 2D,E presented that there was significantly increased neuronal death in the hippocampus of mice in the CA/CPR group, manifested by deeply stained shrunken pyramidal cells with enlarged intercellular spaces. Consistent with the results of behavior tests, AA147 significantly reduced the amounts of dead neurons in the hippocampal cornu ammonis (CA1) region. Likewise, FJB staining showed much more surviving neurons in the hippocampus of AA147-treated mice, indicated by markedly fewer FJB-positive cells (Figures 2F,G). Moreover, we evaluated weight loss and serum aspartate aminotransferase (AST) levels in mice (Supplementary Figure S1). There were no significant differences among the groups. The current results, coupled with the previous study, illustrate no apparent toxic side effect of AA147 (Blackwood et al., 2019). Collectively, our data indicate the neuroprotective effects of AA147 after CA/CPR.

### AA147 alleviated neuronal apoptosis caused by post-CA/CPR cerebral IRI

**FIGURE 3:**
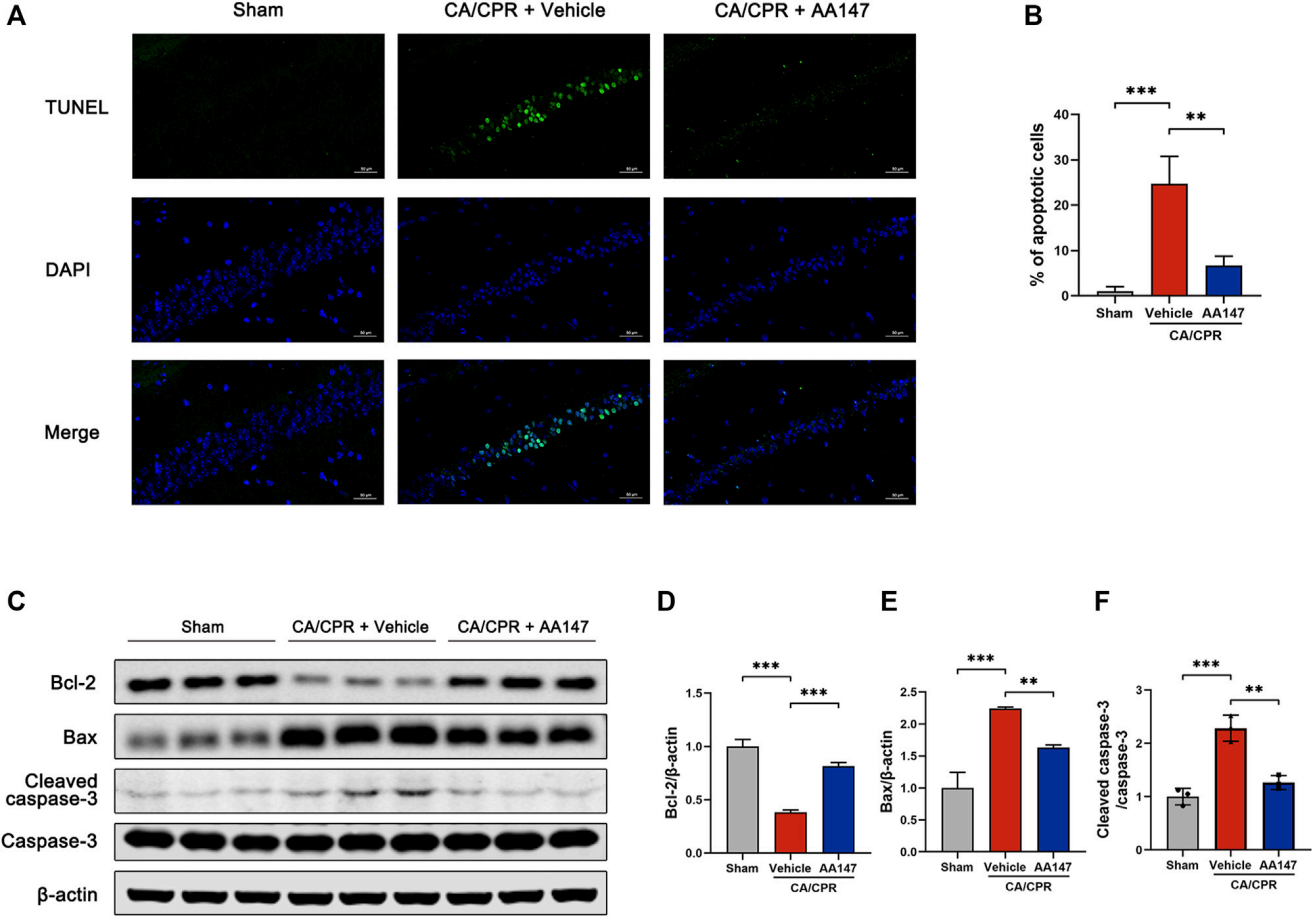
AA147 alleviated neuronal apoptosis induced by cardiac arrest and cardiopulmonary resuscitation (CA/CPR). Mice were subjected to sham or CA/CPR surgery. The brain tissue samples were collected for the TUNEL assay at 3 days after CA/CPR, and the hippocampus of mice that were sacrificed at 1 day after CA/CPR were used for western blot assay. **(A)** TUNEL staining of the hippocampal CA1 subfield and **(B)** quantification of the apoptotic neurons. Numerous TUNEL^+^ cells were observed in the hippocampus of mice in CA + Vehicle group, while treatment with AA147 significantly reduced the counts of TUNEL^+^ cells. Scale bar = 50 μm. **(C)** Western blot assay of apoptosis-related proteins: **(D)** Bcl-2, **(E)** Bax, **(F)** cleaved-caspase-3, and caspase-3. The increased CA/CPR-induced apoptotic signals were reduced by AA147. Data are displayed as mean ± SD (*n* = 3). *p < 0.05. **p < 0.01. ***p < 0.001.

We performed the TUNEL analysis of the hippocampal CA1 area to confirm whether AA147 could counteract post-CA/CPR neuronal apoptosis. As shown in Figures 3A,B, there are plenty of apoptotic cells in the hippocampal CA1 area of mice in CA/CPR + vehicle group, whereas AA147 significantly reduced the apoptotic rate. Moreover, the levels of apoptosis-related proteins were evaluated (Figures 3C–E). Bcl-2 was significantly blunted after CA/CPR, accompanied by increased Bax. In contrast, the AA147-treated group presented an opposite tendency of Bcl-2/Bax. Likewise, cleaved caspase-3, responsible for executing apoptosis, surged markedly after CA/CPR. Still, it was reversed by AA147 (Figures 3C,F). Therefore, AA147 could protect cerebral tissue from IRI post-CA by suppressing apoptosis.

### AA147 restrained oxidative stress and suppressed ER stress in mouse brain after CA/CPR

**FIGURE 4:**
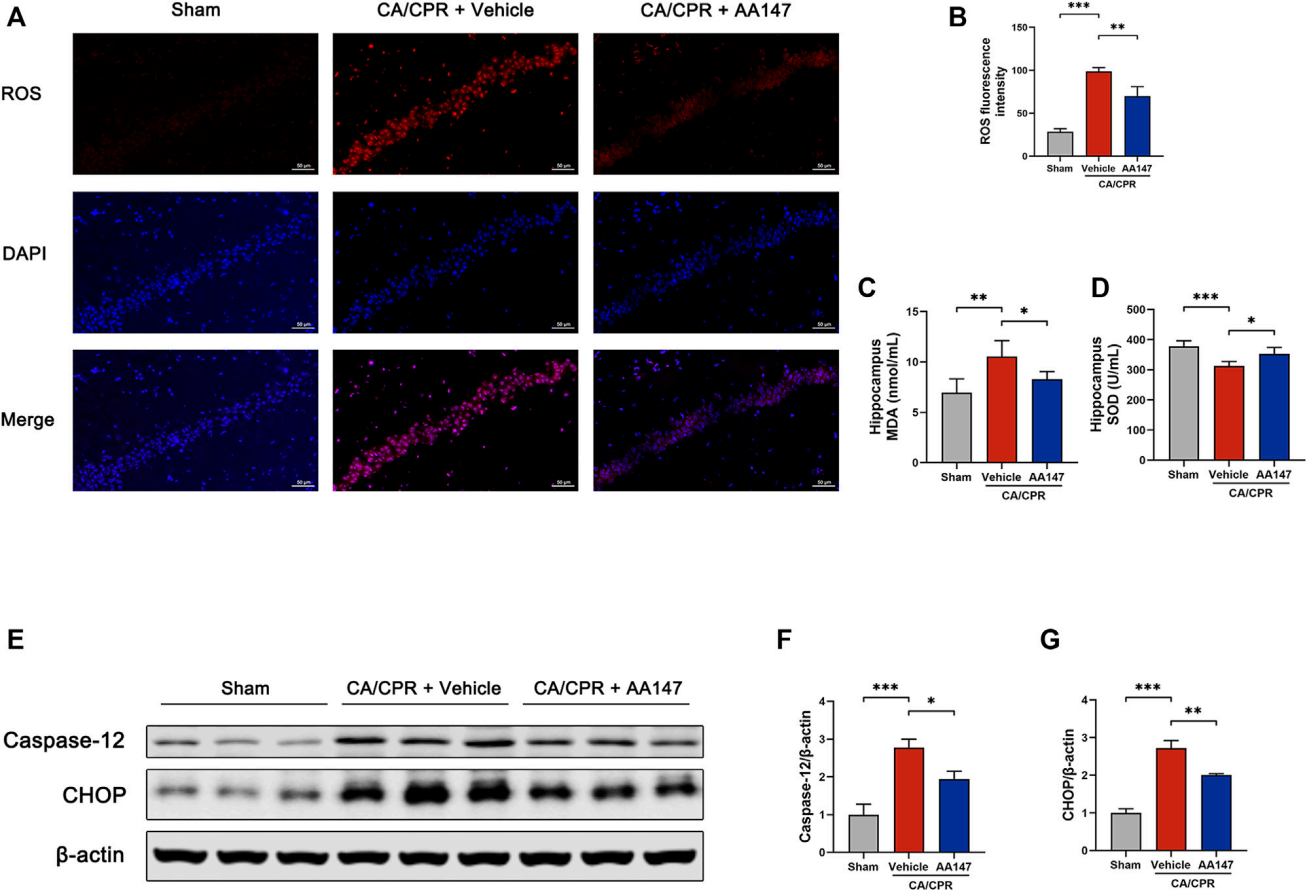
AA147 suppressed post-cardiac arrest and cardiopulmonary resuscitation (CA/CPR) oxidative stress and ER stress. Mice in each group were sacrificed at 1 day after CA/CPR to assess the effect of AA147 on oxidative stress and ER stress induced by post-CA/CPR cerebral ischemia/reperfusion. **(A)** DHE staining was used to measure ROS production in the hippocampal CA1 region. Scale bar = 50 μm. **(B)** Quantification of ROS fluorescence intensity showed that AA147 reduced the overproduction of ROS caused by CA/CPR. Data are displayed as mean ± SD (*n* = 3) **(C)** The MDA and **(D)** SOD activity levels of the hippocampal tissue were detected by commercially available kits. Data are displayed as mean ± SD (*n* = 5). **(E)** Western blot analysis of ER stress-associated proteins: **(F)** caspase-12 and **(G)** CHOP. Data are displayed as mean ± SD (*n* = 3). *p < 0.05. **p < 0.01. ***p < 0.001.

To further explore the underlying mechanism of AA147-dependent protective effect on the brain after CA/CPR, we focused on neuronal ER stress and oxidative stress resulting from CA/CPR-induced cerebral ischemia/reperfusion. ROS production of the hippocampus was measured by DHE staining, as presented in Figures 4A,B. CA/CPR significantly stimulated ROS generation in the hippocampus, mitigated by AA147 administration. Similarly, the MDA concentration in hippocampal homogenate in AA147 treated group decreased compared with CA/CPR + vehicle group (Figure 4C), while the SOD activity was significantly reinforced (Figure 4D). Concerning the levels of ER stress, we detected the expression of caspase-12 and C/EBP homologous protein (CHOP), the biomarker of the terminal arm of UPR, indicating apoptosis induced by ER stress. Caspase-12 and CHOP protein levels, which increased after CA/CPR, were considerably repressed by AA147, as shown in Figures 4E–G. Therefore, CA/CPR-mediated cerebral oxidative stress and ER stress were considerably relieved by AA147.

### AA147 activated the ATF6 and Nrf2 signaling pathways in mouse brain

**FIGURE 5:**
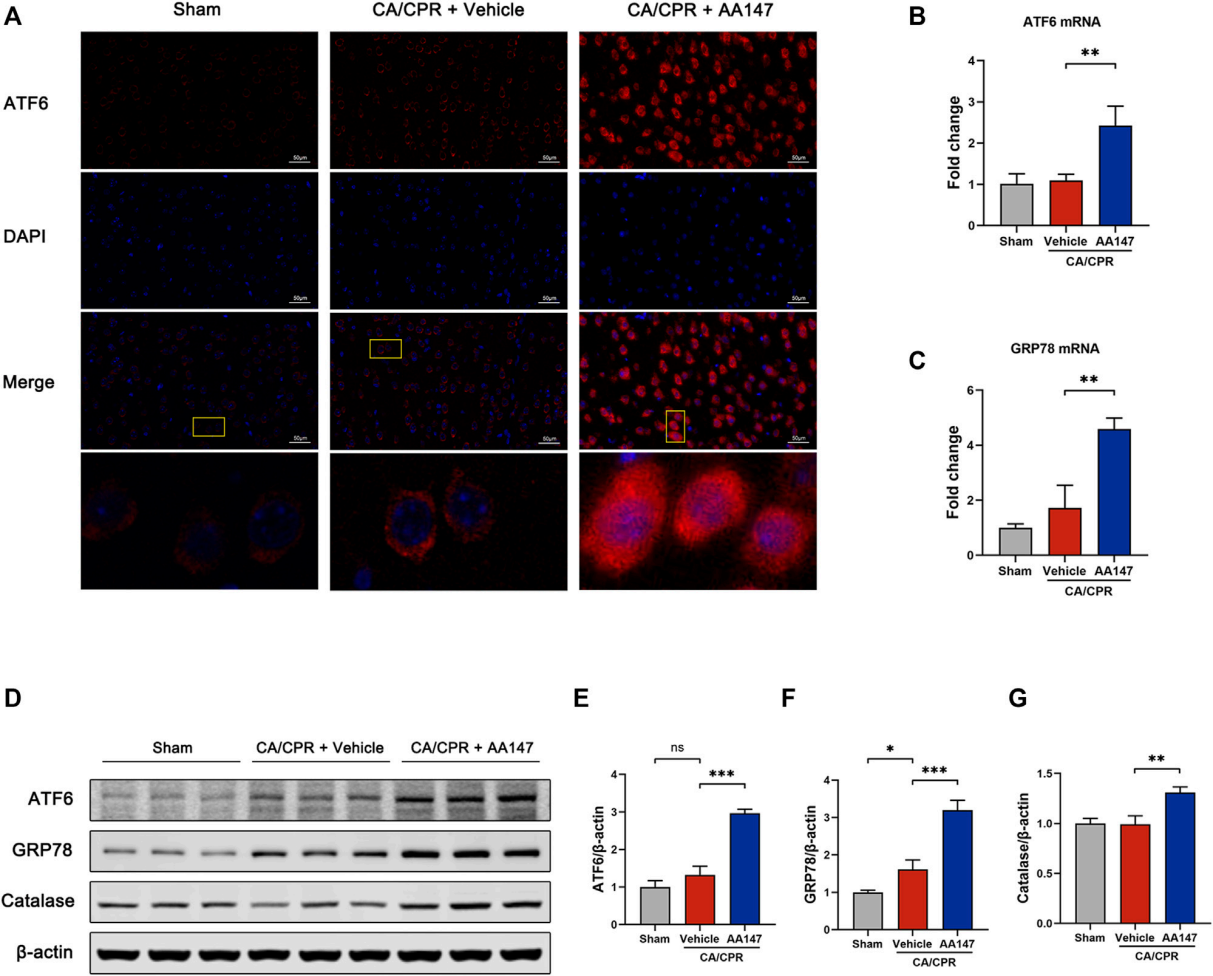
ATF6 signaling pathway was activated by AA147 in neurons after cardiac arrest and cardiopulmonary resuscitation (CA/CPR). The brain tissue samples of mice in each group were collected at 1 day after CA/CPR to determine whether the ATF6 signaling is activated by AA147. **(A)** Representative images of immunofluorescent staining of ATF6 (red) showed that AA147 promoted nuclear translocation of ATF6. Scale bar = 50 μm. **(B)** The mRNA expression of ATF6 and **(C)** its downstream gene GRP78 were determined by qPCR. **(D)** Western blot analysis of **(E)** ATF6, **(F)** GRP78, and **(G)** catalase protein levels in the hippocampus of each group. Data are displayed as mean ± SD (n = 3). *p < 0.05. **p < 0.01. ***p < 0.001.

AA147 was initially reported to be a specific activator for the ATF6 branch of UPR (Plate et al., 2016). Consistent with prior studies, our findings proved the upregulated expression of ATF6 and its modulated genes, which is observed in Figure 5. Immunofluorescence showed that surged ATF6 translocated to the nucleus in neurons with AA147 treatment (Figure 5A). CA/CPR modestly elevated the mRNA and protein levels of ATF6 but without significant differences, while AA147 administration further significantly increased ATF6 expression (Figures 5B,D,E). Glucose-regulated protein 78 (GRP78) is an ATF6-regulated gene, the elevation of which indicates the activation of ATF6. Herein, the mRNA and protein levels of GRP78 increased in CA/CPR versus the sham group and were further strengthened through AA147 treatment (Figures 5C,D,F). In addition, AA147 also elevated the protein level of catalase, which was previously known to be regulated by ATF6 (Figures 5D,G). Combined, AA147 was an effective pharmacological tool to boost the ATF6 signaling pathway.

**FIGURE 6:**
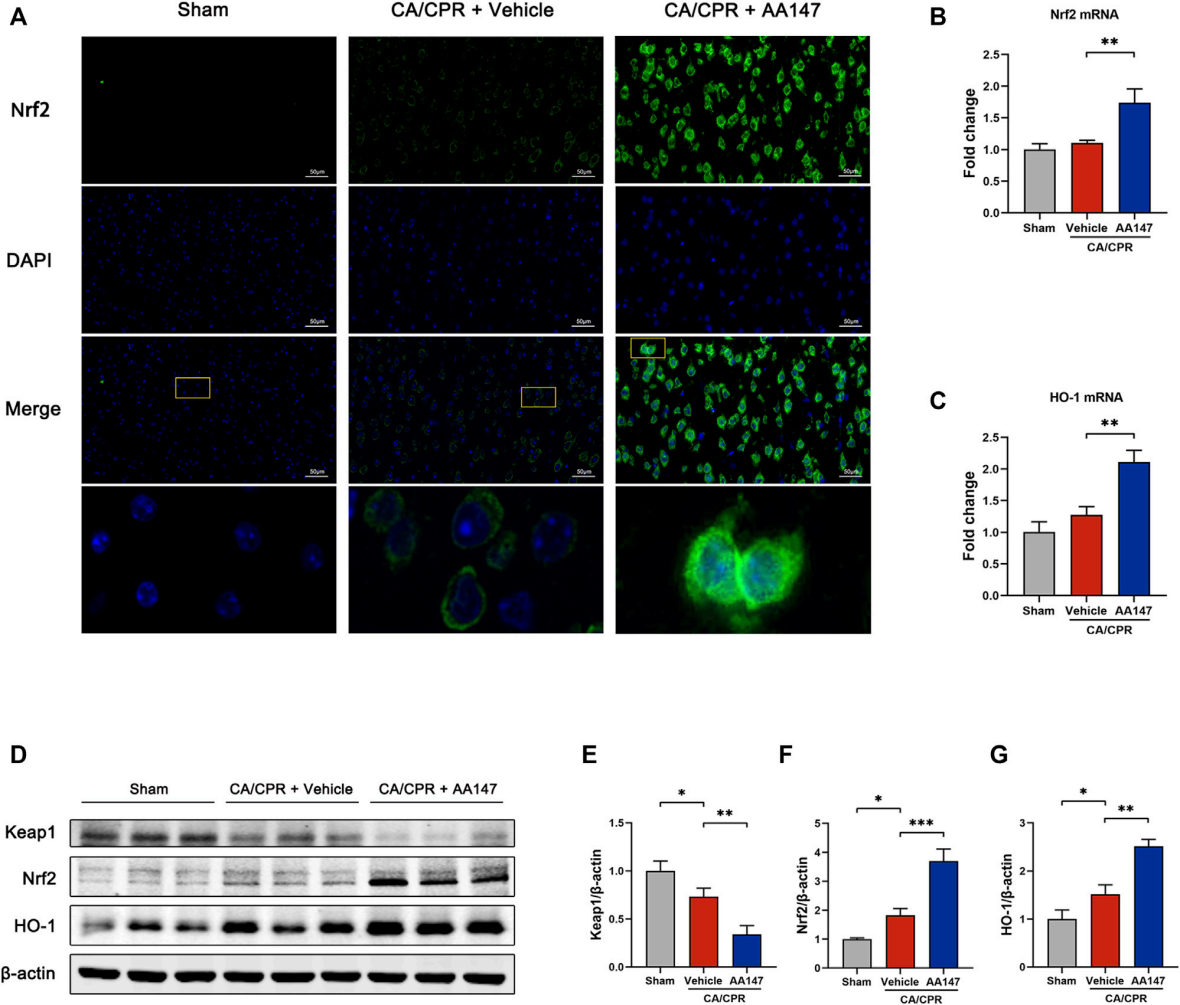
Nrf2 signaling pathway was activated by AA147 in neurons after cardiac arrest and cardiopulmonary resuscitation (CA/CPR). The brain tissue samples of mice in each group were collected at 1 day after CA/CPR to detect the AA147-dependent activation of Nrf2. **(A)** Representative images of immunofluorescent staining of Nrf2 (green) presented that AA147 elevated the expression of Nrf2 and increased its nuclear accumulation. Scale bar = 50 μm. **(B)** The mRNA levels of Nrf2 and **(C)** HO-1 were examined by qPCR. **(D)** Western blot analysis of **(E)** Keap1, **(F)** Nrf2, and **(G)** HO-1. Data are displayed as mean ± SD (*n* = 3). *p < 0.05. **p < 0.01. ***p < 0.001.

A recent study elucidates that AA147 also activates Nrf2 through covalent modifying Kelch-like ECH-associated protein 1 (Keap1) (Rosarda et al., 2021). The mechanism is similar to that of AA147-dependent ATF6 activation. Nrf2 signaling is regarded as an essential modulator in the anti-oxidative process. We sought to evaluate the activation of the Nrf2 signaling pathway by AA147. Immunofluorescent staining showed a remarkable increase and the nuclear translocation of Nrf2 (Figure 6A). Keap1 protein level was downregulated when treated by AA147 in CA/CPR, while Nrf2 and its downstream heme oxygenase-1 (HO-1) were remarkably enhanced at protein and mRNA levels (Figures 6B–G). Intriguingly, the Nrf2 signal pathway was also slightly increased after CA/CPR with merely a control vehicle, but it failed to have a significant protective effect. Collectively, the substantial activation of Nrf2 signaling implied that it contributed to AA147-dependent cerebral protection to a certain extent.

### Pharmacologic ATF6 and Nrf2 inhibition reduces AA147-dependent protection against post-CA/CPR cerebral IRI

**FIGURE 7:**
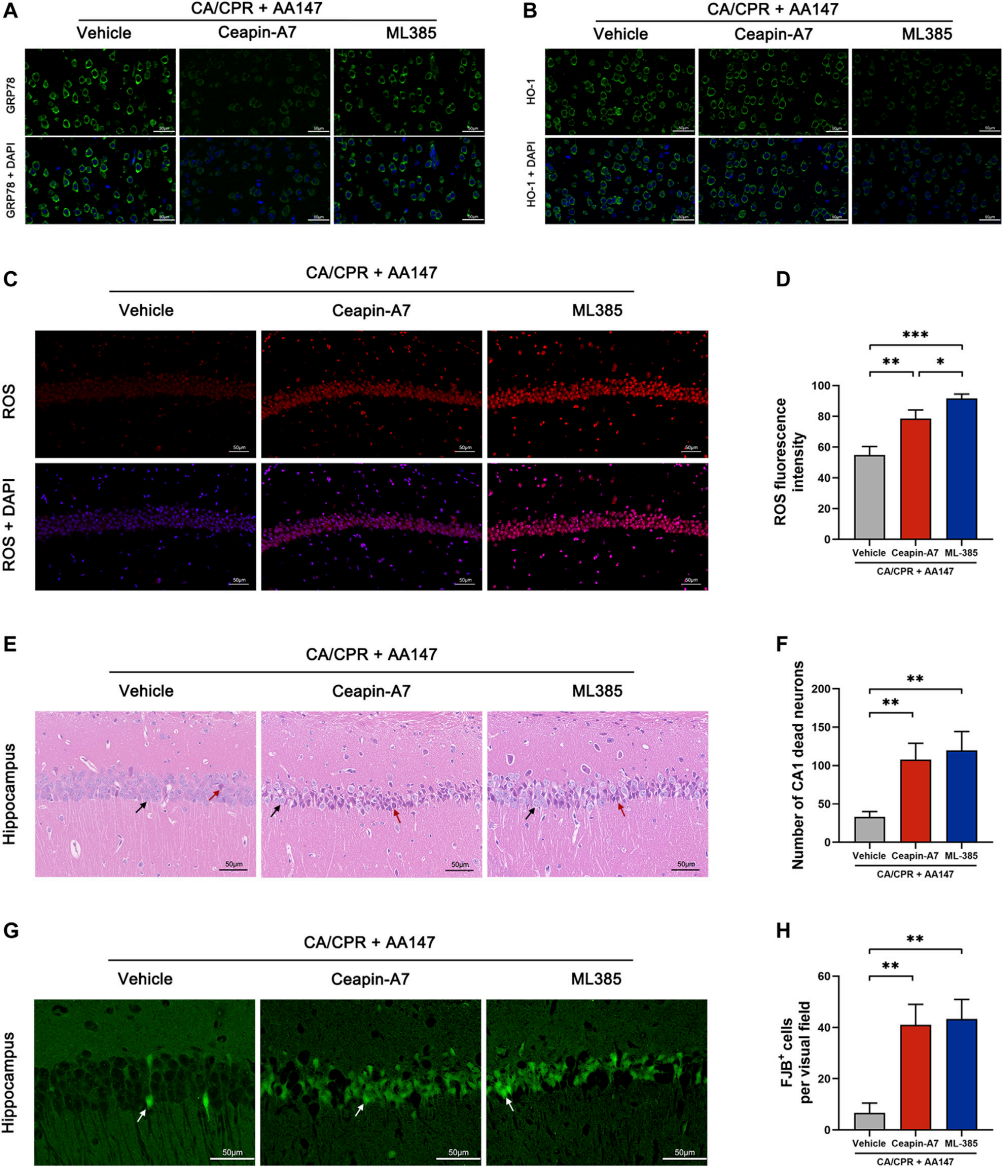
Protective effect of AA147 against post-cardiac arrest cerebral ischemia/reperfusion injury was abolished by the ATF6 inhibitor Ceapin-A7 or the Nrf2 inhibitor ML385. Ceapin-A7 or ML385 were administered to mice 2 h before the administration of AA147. The brain tissue samples were collected for immunofluorescent staining and ROS detection at 1 day after CA/CPR and for evaluation of neuronal death at 3 days after CA/CPR. **(A)** Representative images of immunofluorescent images of GRP78 showed that Ceapin-A7 decreased AA147-induced increase in ATF6-regulated gene GRP78 expression. **(B)** Representative images of HO-1 presented that AA147-induced increase in Nrf2-regulated gene expression was inhibited by ML385 but not affected by ATF6 inhibitor Ceapin-A7. **(C)** Representative images of DHE staining in the hippocampus and **(D)** quantification of ROS fluorescent intensity showed that there was much more ROS overproduction in the hippocampus of mice in either AA147+Ceapin-A7 or AA147 + ML385 group compared with mice only treated with AA147. **(E)** Representative images of HE staining in hippocampal CA1 area and **(F)** number of CA1 dead neurons (black arrow: normal neuron; red arrows: dead neuron). **(G)** Representative images of FJB staining in hippocampal CA1 area (white arrow: FJB^+^ neuron) and **(H)** counts of FJB^+^ cells. Plenty of neuronal death was observed in the hippocampus of mice injected with the ATF6 inhibitor Ceapin-A7 or the Nrf2 inhibitor ML385, even with AA147 administered follow by. Scare bar = 50 μm. Data are displayed as mean ± SD (*n* = 3). *p < 0.05. **p < 0.01. ***p < 0.001.

To determine the role of ATF6 and Nrf2 in AA147-dependent protection against post-CA cerebral IRI, we treated mice with both AA147 and small molecular inhibitors of ATF6 or Nrf2. Ceapin-A7 could block the transformation of the ATF6 oligomer to monomer and its release from ER to the cytoplasm (Gallagher and Walter, 2016). ML385 inhibits Nrf2 transcription by affecting its DNA binding (Singh et al., 2016). As shown in Figures 7A,B, Ceapin-A7 impeded the AA147-dependent increase in GRP78, ATF6-regulated gene, but not HO-1, which is modulated by Nrf2. ML385 blocked the surge of HO-1, while GRP78 was still elevated by AA147. As shown in Figures 7C,D, the reduction in AA147-dependent ROS scavenging was observed in the absence of ATF6 or Nrf2 activation. However, the ROS accumulation was more evident in neurons with Nrf2 inhibited by ML385 before AA147 treatment compared to the Ceapin-A7 group. It implied that ATF6 activation contributed less to ROS removal but might play a role in AA147-dependent neuroprotection mainly by modulating ER proteostasis. Moreover, as illustrated with HE and FJB staining (Figures 7E–H), there are many more dead neurons in the hippocampus of mice treated with Ceapin-A7 or ML385 before the administration of AA147, indicating that the AA147-dependent neuroprotection was partly abolished with either the ATF6 or the Nrf2 inhibited. Combined, these results suggested that the neuroprotection afforded by AA147 against post-CA cerebral IRI was mediated by the co-regulation of ATF6 and Nrf2 activity.

## Discussion

**FIGURE 8:**
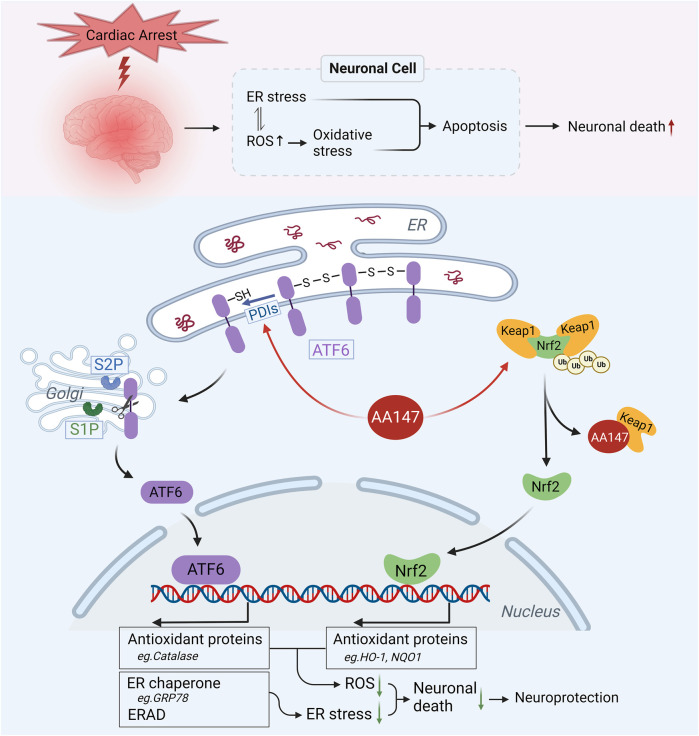
Proposed mechanism for the neuroprotective effect of AA147 against post-cardiac arrest (CA) cerebral ischemia/reperfusion injury (IRI). ER stress and oxidative stress induced by post-CA cerebral IRI results in numerous neuronal death. AA147 elevates ATF6 and Nrf2 levels in the neurons and increases their nuclear translocation by covalently modifying PDIs (restraining ATF6 within ER by regulating ATF6 disulfide) and Keap1 (keeping Nrf2 in low levels outside the nucleus by promoting its ubiquitination). AA147-induced activation of ATF6 could upregulate the expression of ER chaperones (e.g., GRP78) and ER-associated protein degradation (ERAD) components to restore ER proteostasis, also activating several antioxidant genes, including catalase, to scavenge overproduced ROS. At the same time, Nrf2 could induce the expression of HO-1, NQO1, and other antioxidant proteins to inhibit oxidative stress. The AA147-induced synergistic effect of two signaling pathways reduces neuronal death caused by post-CA cerebral IRI.

In the present study, we showed that: 1) AA147 significantly ameliorated post-CA/CPR cerebral IRI, reducing CA/CPR-induced neuronal death and improving neuro-functional rehabilitation. 2) AA147 exhibited neuroprotective effects by suppressing oxidative stress, ER stress, and apoptosis. 3) The protection afforded by AA147 against cerebral IRI was mediated by the co-modulation of adaptive ATF6 and Nrf2 signaling pathways. (Figure 8).

CA/CPR triggers global IRI in which neuron is the most vulnerable. IRI has been shown to disrupt the protein homeostasis of the ER in neurons (Yang and Paschen, 2016). Increasing evidence indicates that modulating the adaptive unfolded protein response to reestablish ER proteostasis protects neurons efficaciously against IRI (Yu et al., 2017; Wang Y.-C. et al., 2020; Li et al., 2021; Wang et al., 2021). AA147, a proteostasis regulator compound, was shown to protect against IRI in multiple disease models. Previous research revealed that AA147 preserved cardiac function after myocardial infarction and protected kidneys and brains against IRI (Blackwood et al., 2019). AA147 is a desirable pharmacologic agent with no apparent side effects, capable of crossing the blood-brain barrier to function in the brain (Blackwood et al., 2019). A recent study proved that AA147 treatment considerably improved CA outcomes, including survival rate and neurological function recovery (Shen et al., 2021). Paralleling these findings, we also observed markedly increased neurological scores and much better neurobehavioral performance of AA147-treated mice after CA/CPR. Moreover, we further elucidated that AA147 treatment in CA/CPR directly protected neurons in the hippocampus from degeneration and death caused by post-CA/CPR IRI. HE, FJB staining, and the TUNEL assay showed considerably reduced dead neuron counts and decreased apoptotic rate. Collectively, these results implied that AA147 represented therapeutic potential in treating post-CA brain injury.

Oxidative stress, appearing along with ROSC after prolonged global ischemia caused by CA, exacerbates post-CA organic IRI (Granger and Kvietys, 2015). Overproduction of ROS results in lipid peroxidation and DNA insults, accelerating cell death (Becker, 2004; Granger and Kvietys, 2015). ROS homeostasis is requisite for oxidative protein folding in the ER. Once the redox homeostasis of the ER is destroyed by excessive ROS generation, misfolded protein will accumulate in the ER then the ER stress occurs (Malhotra and Kaufman, 2007; Zhang et al., 2019). ER redox state is inseparable from ER proteostasis, showing synergism with each other in the normal functioning of ER. In the present study, our results showed that oxidative stress and ER stress levels were upregulated in the hippocampus when mice were subjected to CA/CPR. An increase in apoptosis-related protein levels, accompanied by surged caspase-12 and CHOP protein expression, indicates ER stress-associated cell apoptosis caused by post-CA/CPR cerebral IRI. It implied that the endogenous adaptive UPR fails to sufficiently restore the ER proteostasis and shifts to the cell death-oriented maladaptive UPR, involving PERK-ATF4-caspase12-CHOP and IRE1-apoptosis signals (Han et al., 2013; Hetz and Papa, 2018; Ren et al., 2021). AA147 was shown to reduce oxidative stress-induced toxicity by decreasing ROS-associated damages in cardiomyocytes or neuronal-derived cells (Blackwood et al., 2019; Rosarda et al., 2021). We also observed that ROS production and MDA levels were reduced markedly by AA147 in neurons after CA/CPR, indicating the effects of AA147 on attenuating oxidative stress. Combined, the results demonstrated that inhibition of oxidative stress and ER stress-associated apoptosis contributes to the AA147-dependent protection against post-CA cerebral IRI.

ATF6, one of the primary sensors of ER stress, is anchored at the ER membrane as disulfide-linked oligomers in stable conditions. In response to ER stress, the ATF6 monomers dissociate from the oligomers after the disulfide bonds are reduced, trafficking to the Golgi and being proteolyzed into the active form that migrates to the nucleus and promotes the expression of stress-responsive genes as a transcription factor (Glembotski et al., 2019). The activated ATF6 can upregulate canonical ER stress response genes that encode ER chaperones (e.g., GRP78) and ER-associated protein degradation (ERAD) components (Martindale et al., 2006; Shen et al., 2021). In addition, ATF6 also plays non-canonical roles in response to ER stress, such as activating several antioxidant genes, including catalase, to scavenge overproduced ROS (Jin et al., 2017; Glembotski et al., 2020). The catalase functions in peroxisomes instead of an ER-resident protein, neutralizing a part of ROS caused by ischemia/reperfusion. Activation of ATF6 has been reported beneficial to multiple tissues suffering from IR injury (Glembotski et al., 2019). Cardiac functions of ATF6 knocked down rats deteriorated, which was reversed by ATF6 overexpression (Jin et al., 2017). Likewise, Yang’s research group used mice with the ATF6 pathway specifically activated in forebrain neurons to indicate the efficacy of ATF6 in improving ischemic stroke and CA outcomes, highlighting the specific roles of ATF6 in neuroprotection (Yu et al., 2017; Shen et al., 2021). While accumulating evidence showed the therapeutic potential of the ATF6 branch to minimize reperfusion damage, the development of more readily implemented therapeutic interventions to apply it to the clinic is highly necessary.

AA147 is a small molecule compound initially developed to preferentially activate the AFT6 signaling, facilitating the rehabilitation of ER proteostasis (Plate et al., 2016). After metabolically activated into a reactive electrophile in the cell, AA147 activates ATF6 through covalent modifications of disulfide isomerases (PDIs), the ER-resident proteins which are necessary for regulating disulfide bonds during ATF6 activation (Paxman et al., 2018). A prior study shows that the AA147-dependent improvement of ER proteostasis and reduction of oxidative stress were counteracted in the absence of ATF6 in IR-treated cardiomyocytes (Blackwood et al., 2019). ATF6 and its modulated genes were maximally activated 24 h after injection of AA147 (Blackwood et al., 2019), so in this study, we treated mice with AA147 at 24 h before CA/CPR for its full effectiveness. As expected, our results showed a significant increase in ATF6 protein levels and expression of its targeted gene GRP78 in neurons of AA147-treated mice after CA/CPR, indicating the activation of the adaptive ATF6 pathway. However, the ATF6 pathway was also induced in CA/CPR + vehicle group. Still, there remained a remarkable difference with the AA147 treated group, suggesting AA147 activated the remaining inactive ATF6 in neurons subjected to ischemia/reperfusion strike for therapeutic reverse. The results also showed that activating ATF6 by AA147 enhanced catalase protein levels in neurons of mice CA/CPR model, contributing to ROS scavenging in part. Moreover, Cotreatment with Ceapin-A7, a compound inhibiting ATF6 activation through stabilizing ATF6 oligomers, blocked the AA147-dependent reduction in neuronal cell death and partly attenuated ROS scavenging. Collectively, the results demonstrated that activation of ATF6 contributes to AA147-dependent neuroprotection against post-CA IRI, probably through helping restore ER proteostasis and to a certain extent remove ROS.

Nrf2 and its induced pathway are the dominant defense mechanism against oxidative stress, offering a pro-survival role by inducing downstream genes that encode HO-1, NAD(P)H: quinone oxidoreductase 1 (NQO1), and other antioxidant proteins once evoked by destructive stressors. Keap1, the principal negative regulator of Nrf2, sustain the Nrf2 in low levels outside the nucleus by promoting its degradation under normal condition (Canning et al., 2015; Suzuki and Yamamoto, 2015; Yamamoto et al., 2018). Extensive research has established that activation of the Nrf2 signaling pathway exhibits neuroprotective effects against cerebral IRI (Sivandzade et al., 2019; Wang P. et al., 2020; Yang et al., 2022). AA147 is reported to provide protective effects independent of ATF6 in several cell types (Almasy et al., 2021; Rius et al., 2021; Rosarda et al., 2021). A recent study shows that AA147 covalently modifies Keap1 to decrease the degradation of Nrf2 and promote its activation, mitigating oxidative toxicity in an Nrf2-dependent manner in neuronal-derived cells (Rosarda et al., 2021). In the present study, we first confirmed an enhancement of Nrf2-regulated antioxidant response by AA147 in mice undergoing CA/CPR. Keap1 protein levels were reduced while the Nrf2 and downstream HO-1 expression was remarkably elevated by AA147. Furthermore, AA147-dependent attenuation of neuronal death and ROS overproduction was significantly reversed by ML385, the inhibitor of Nrf2. Considering that the ROS excess was more significant after the inhibition of Nrf2 than the ATF6, AA147-dependent ROS reduction may be primarily mediated by Nrf2 activation.

The present study has several limitations. Firstly, the mouse’s body temperature, which is supposed to cool down over time naturally, was artificially maintained at around 37°C during CA/CPR, which may weaken the clinical significance. However, it is necessary to eliminate interference from hypothermia to investigate the neuroprotective effect of AA147. Because it is well-established that hypothermia confers cerebral protection from CA/CPR (Nolan et al., 2021). Secondly, the pretreatment dose of AA147 was administered at 1 day before CA, which is far from clinical practice. CA is a sudden acute emergency with prior-treatment approaches unrealistic. Further studies are demanded to validate the beneficial effect of posttreatment with AA147 on CA outcomes. Moreover, we merely evaluated the impact of AA147 in the time point of 1 and 3 days after resuscitation. More and longer time points for detection, like 7 and 14 days after ROSC, are needed to verify the prolonged treatment effects of AA147 for CA. Thirdly, worse post-CA neurological outcomes comes to aged mice (Shen et al., 2018), so the proposed drug dose of AA147 should vary in different age groups. The effects of AA147 on aged mice subjected to CA are also necessary to be elucidated. Finally, given the evidence that microglia and astroglia play a critical role in cerebral IRI (Peberdy et al., 2016), that ATF6 involves in astrogliosis (Yoshikawa et al., 2015) and microglia-mediated neuroinflammation (Ta et al., 2016), it is necessary to explore more potential mechanisms, such as the effect of AA147 on microglia and astroglia, in the following studies on cerebral resuscitation after CA.

## Conclusion

In summary, our findings demonstrated that AA147-induced activation of the ATF6 and Nrf2 pathways ameliorated post-cardiac arrest cerebral ischemia/reperfusion injury, improving neurological outcomes and reducing neuronal death by suppressing ER stress-associated apoptosis and oxidative stress.

## Data Availability

The original contributions presented in the study are included in the article/Supplementary Material, and further inquiries can be directed to the corresponding author.
