# Supplementary material for: AA147 ameliorates post-cardiac arrest cerebral ischemia/reperfusion injury through the co-regulation of the ATF6 and Nrf2 signaling pathways
Source: Front Pharmacol. 2022 Nov 23;13:1028002. doi: 10.3389/fphar.2022.1028002 (PMC9727236; doi:10.3389/fphar.2022.1028002)
Supplement: Supplementary file 2 [file Image1.pdf]

Supplementary Material

Supplementary Figures

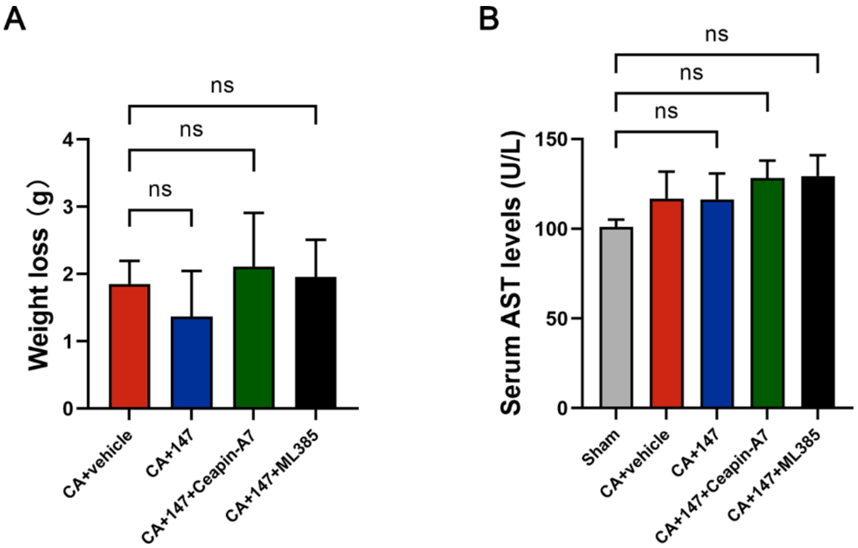

Supplementary Figure 1. AA147, Ceapin-A7 and ML385 did not cause toxic side effects in mice post-cardiac arrest and cardiopulmonary resuscitation (CA/CPR). (A) Weight loss among groups at 1 day after CA/CPR. (B) Serum levels of aspartate amino transferase (AST) were measured at 1 day after CA/CPR. Data are presented as mean ± SD (n=3 mice per group), *P<0.05. **P<0.01. ***P<0.001.
